# ^177^Lu-NM600 Targeted Radionuclide Therapy Extends Survival in Syngeneic Murine Models of Triple-Negative Breast Cancer

**DOI:** 10.2967/jnumed.119.236265

**Published:** 2020-08

**Authors:** Reinier Hernandez, Joseph J. Grudzinski, Eduardo Aluicio-Sarduy, Christopher F. Massey, Anatoly N. Pinchuk, Ariana N. Bitton, Ravi Patel, Ray Zhang, Aakarsha V. Rao, Gopal Iyer, Jonathan W. Engle, Jamey P. Weichert

**Affiliations:** 1 Department of Radiology, University of Wisconsin–Madison, Madison, Wisconsin; 2 Department of Medical Physics, University of Wisconsin–Madison, Madison, Wisconsin; 3 Department of Human Oncology, University of Wisconsin–Madison, Madison, Wisconsin; and; 4 UW Carbone Cancer Center, University of Wisconsin–Madison, Madison, Wisconsin

**Keywords:** ^177^Lu-NM600, triple-negative breast cancer, TNBC, targeted radionuclide therapy, theranostics

## Abstract

There is a clinically unmet need for effective treatments for triple-negative breast cancer (TNBC), as it remains the most aggressive subtype of breast cancer. Herein, we demonstrate a promising strategy using a tumor-targeting alkylphosphocholine (NM600) for targeted radionuclide therapy of TNBC. **Methods:** NM600 was radiolabeled with ^86^Y for PET imaging and ^177^Lu for targeted radionuclide therapy. ^86^Y-NM600 PET imaging was performed on female BALB/C mice bearing syngeneic 4T07 (nonmetastatic) and 4T1 (metastatic) TNBC tumor grafts (*n* = 3–5). Quantitative data derived from a PET-image region-of-interest analysis, which was corroborated by ex vivo biodistribution, were used to estimate the dosimetry of ^177^Lu-NM600 treatments. Weight measurement, complete blood counts, and histopathology analysis were performed to determine ^177^Lu-NM600 toxicity in naïve BALB/C mice administered 9.25 or 18.5 MBq. Groups of mice bearing 4T07 or 4T1 grafts (*n* = 5–6) received excipient or 9.25 or 18.5 MBq of ^177^Lu-NM600 as a single or fractionated schedule, and tumor growth and overall survival were monitored. **Results:** Excellent tumor targeting and rapid normal-tissue clearance of ^86^Y-NM600 were noted in both 4T07 and 4T1 murine models. Ex vivo biodistribution corroborated the accuracy of the PET data and validated ^86^Y-NM600 as a surrogate for ^177^Lu-NM600. ^177^Lu-NM600 dosimetry showed absorbed doses of 2.04 ± 0.32 and 1.68 ± 0.06 Gy/MBq to 4T07 and 4T1 tumors, respectively, which were larger than those delivered to liver (1.28 ± 0.09 Gy/MBq) and to bone marrow (0.31 ± 0.05 Gy/MBq). The ^177^Lu-NM600 injected activities used for treatment were well tolerated and resulted in significant tumor growth inhibition and prolonged overall survival in both tested TNBC models. A complete response was attained in 60% of treated mice bearing 4T07 grafts. **Conclusion:** Overall, our results suggest that ^177^Lu-NM600 targeted radionuclide therapy has potential for TNBC and merits further exploration in a clinical setting.

Breast cancer accounted for 30% of the almost 880,000 new cancer cases in 2018 in the United States (1). Approximately 15%–20% of all cases are considered triple-negative breast cancer (TNBC) because of undetectable levels of estrogen receptor, progesterone receptor, and human epidermal growth factor receptor 2 (HER2) protein at the time of diagnosis. The incidence of TNBC is higher in young minority women, who face a worse prognosis (both higher rates of early recurrence and higher rates death from their disease) than other ethnic groups (2,3). Inferior outcomes for TNBC are, in large part, attributable to the common presence of micrometastatic disease at diagnosis and the absence of efficacious, molecularly targeted therapies such as the antiestrogenic aromatase inhibitors for estrogen receptor–positive breast cancer and the HER2-directed agents such as trastuzumab for breast cancers expressing high (3+ score) levels of HER2. Therapies currently used to treat clinically occult TNBC micrometastatic disease are limited to conventional cytotoxic chemotherapies (e.g., anthracyclines and taxanes) that have modest efficacy at the expense of severe toxicities (4).

Metabolically stable alkylphospholipid derivatives, including alkylphosphocholines, are structural mimics of cell membrane phospholipids that selectively accumulate in glycosphingolipid- and cholesterol-rich cellular membrane microdomains, known as lipid rafts (5,6). Lipid rafts, which play important roles in cancer cell proliferation, survival, and metastasis, are markedly overexpressed by malignant cells compared with normal cells, constituting a promising mechanism for cancer-targeted therapies (7–11). Leveraging such a nearly universal, receptor-independent targeting mechanism, we have developed several alkylphosphocholine analogs displaying selective tumor uptake and retention in a wide variety of cancer types, including breast cancer. In this study, we investigated the properties of NM600, an alkylphosphocholine analog with a desirable tumor selectivity and pharmacokinetic profile for targeted radionuclide therapy (a systemic radiotherapy approach that has succeeded clinically in treating several advanced cancers) in TNBC (12,13). Results show that the PET imaging agent ^86^Y-NM600 selectively targets murine TNBC tumors and can serve as a PET imaging surrogate to model the distribution and estimate the dosimetry of the therapeutic congener ^177^Lu-NM600. In therapeutic studies using ^177^Lu-NM600, 4T07 and 4T1 murine cancers were used to investigate 2 clinically relevant TNBC phenotypes: locally aggressive and highly metastatic, respectively (14). In these models, treatment with ^177^Lu-NM600 afforded tumor control and extended overall survival while showing a favorable dosimetry and toxicity profile that merits further exploration of this agent in human subjects.

## MATERIALS AND METHODS

### Radiochemistry and Stability

^86^Y-chloride was provided by the University of Wisconsin–Madison cyclotron group (15), and ^177^Lu chloride was purchased from the Missouri University Research Reactor. Radiolabeling of 2-(trimethylammonio)ethyl(18-(4-(2-(4,7,10-tris(carboxymethyl)-1,4,7,10-tetraazacyclododecan-1-yl)acetamido)phenyl)octadecyl) phosphate (NM600) with ^86^Y and ^177^Lu proceeded by mixing 185–370 MBq (5–10 mCi) of the radiometal with 55–110 nmol (50–100 μg) of NM600 in 0.5 M NaOAc buffer (pH 5.5). The reaction was then incubated at 90°C for 30 min under constant shaking (500 rpm). ^86^Y/^177^Lu-NM600 was purified by reverse-phase chromatography using Sep Pak C18 cartridges (Waters), eluted in absolute ethanol, dried under a N_2_ stream, and reconstituted in an excipient consisting of normal saline containing 0.4% v/v polysorbate 20. Radiochemical yield was assessed by thin-layer chromatography using a 50 mM ethylenediaminetetraacetic acid mobile phase, which moves the free radiometals with the solvent front (R_f_ = 1) while ^86^Y/^177^Lu-NM600 remains at the origin (R_f_ = 0).

Radiochemical purity and stability were determined via radiolabeled high-performance liquid chromatography (HPLC) using a reverse-phase 250 × 3.00 mm C18 Luna 5 μm 100 Å column (Phenomenex) and a water:acetonitrile gradient (5% MeCN: 0–2 min; 5%–65% MeCN: 2–30 min; 65%–90% MeCN: 30–35 min; 90%–5% MeCN: 35–45 min). To assess stability, 9.25 MBq of ^177^Lu-NM600 were incubated at 37°C in both excipient and whole mouse serum, and samples were analyzed by HPLC after 2, 24, 48, 120, or 192 h of incubation. Serum samples were first mixed with an equal volume of MeCN to precipitate serum proteins and then centrifuged, and the supernatant was injected into the HPLC system. All visible peaks were integrated to determine both the initial purity and the stability of ^177^Lu-NM600.

### Cell Culture and Animal Models

Murine mammary adenocarcinoma 4T1 and 4T07 cells lines, which differ in their metastatic capacity but share a common origin (14), were obtained from ATCC. The cells were cultured in RPMI 1640 complete medium supplemented with 10% fetal bovine serum and 1% penicillin/streptomycin in an incubator at 37°C and a 5% CO_2_ atmosphere.

All animal experiments were performed with the approval of the University of Wisconsin Institutional Animal Care and Use Committee. Tumor grafts were induced by subcutaneous injection of 5.0–7.5 × 10^5^ 4T1 or 4T07 cells into the right lower flank of 8-wk-old female BALB/C mice (Envigo). Tumor-bearing mice were used for in vivo imaging and therapy studies approximately 2 wk after implantation, when tumor volume reached 400 mm^3^_._

### ^86^Y-NM600 PET/CT Imaging

For noninvasive in vivo PET/CT imaging, the mice (*n* = 3) were administered 9.25 MBq (250 μCi) of ^86^Y-NM600 via lateral tail-vein injection. After isoflurane anesthesia (4% induction; 2% maintenance), the mice were placed prone into the bore of a small-animal PET/CT scanner (Inveon; Siemens) and static PET scans with 40–80 million coincidence events were acquired at 3, 24, 48, and 72 h after injection of the radiotracer. CT scans were taken before each PET acquisition for attenuation correction and anatomic reference. List-mode PET scans were reconstructed using 3-dimensional ordered-subset expectation maximization. Quantitative analysis of the images was performed by manually drawing volumes of interest over the tumor and other organs of interest, and data were reported as percentage injected activity per gram of tissue (%IA/g, mean ± SD).

### Ex Vivo Biodistribution

Ex vivo tissue distribution studies were performed to corroborate the accuracy of the in vivo imaging quantification results and to test whether ^86^Y-NM600 is a valid PET imaging surrogate for therapeutic ^177^Lu-NM600. Two cohorts of mice bearing 4T07 or 4T1 subcutaneous tumor grafts were administered either 9.25 MBq of ^86^Y-NM600 or 3.7 MBq of ^177^Lu-NM600 via lateral tail-vein injection and sacrificed by CO_2_ asphyxiation 72 h after injection of the tracer. The tumor and other tissues, including blood, heart, liver, lungs, spleen, intestine, pancreas, stomach, bone, and muscle, were collected, wet-weighed, and counted in an automatic γ-counter (Wizard 2; Perkin Elmer). Uptake in each tissue was expressed as %IA/g (mean ± SD).

### Dosimetry Estimations

Imaging and biodistribution results were used in combination with a standardized Monte Carlo N-particle–generated mouse model to estimate ^177^Lu-NM600 dosimetry within the tumor and organs of interest (16,17). The average %IA/g at a given time point (%IA/g(*t*)) within the liver, spleen, kidneys, heart, tumor, bone marrow, and whole body at each time point was extrapolated to the masses of the organs of the mouse model to compute the total injected activity within each source organ (%IA/g_src_(*t*)). Organs that were not delineated were assigned a total injected activity proportional to their organ masses (mass_src_) according to the following equation:

$${\text{\%IA}}_{\text{src}}\left(t\right)=\%{\text{IA}}_{\text{WB}}\left(t\right)\cdot \frac{{\text{mass}}_{\text{src}}}{{\text{mass}}_{\text{body}}},$$

where %IA_WB_/g(*t*) is the %IA within the whole body at a given time point,t. A piecewise time-integral of %IA/g_src_(*t*) was used to derive the cumulative activity within each source organ, Ã_src_:

$${\tilde{\text{A}}}_{\text{src}}=\int_{0}^{\infty }{\text{\%IA}}_{\text{src}}\left(t\right)dt.$$

The standard mouse model, which defines the self-organ and cross-organ energy contributions to target tissue absorbed dose, S(target ← src), converts the cumulative activity within each source organ into the ^177^Lu-NM600 absorbed dose per injected activity (Gy/MBq) within the tumor and each target organ (${\tilde{\text{D}}}_{\text{target}})$ by summing dose contributions from all of the source organs, including the target organ itself:

$${\tilde{\text{D}}}_{\text{target}}=\sum^{}{\tilde{\text{A}}}_{\text{src}}\cdot \text{S}\left(\text{target}\leftarrow \text{src}\right).$$

### Toxicity Evaluation

For longitudinal analysis of complete blood counts, groups of naïve BALB/C mice (*n* = 5) were bled (50 μL) weekly via tail vein nick for 6 wk after injection of nonlabeled NM600, 9.25 MBq, or 18.5 MBq of ^177^Lu-NM600. Complete blood counts were determined with a VetScan HM5 (Abaxis) differential hematology analyzer. After the last bleeding, the mice were sacrificed, and the liver, spleen, kidneys, intestines, and femur were harvested, fixed in formalin, and sectioned for hematoxylin and eosin staining. Stained tissues were examined by a trained pathologist for gross findings.

### ^177^Lu-NM600 Treatment

Mice bearing either nonmetastatic 4T07 or metastatic 4T1 subcutaneous tumors were used for therapy studies when the tumors reached a volume of approximately 400 mm^3^. Groups of 5–10 mice were administered ^177^Lu-NM600 at 2 injected activities, 9.25 MBq (250 μCi) or 18.5 MBq (500 μCi), or an equal mass (2.5 μg) of nonradioactive NM600 (control), via lateral tail-vein injection. Two additional groups of mice bearing the more aggressive 4T1 tumors received 2 ^177^Lu-NM600 fractions (2 × 9.25 MBq or 18.5 + 9.25 MBq) 10 d apart. Animal weight and tumor volume by calipers was monitored 2–3 times per week, and general well-being was assessed daily by the veterinary staff. Humane endpoints were implemented, and animals were retired from the study if significant weight loss (>15% within a week or 20% total) was observed or when tumors reached 700% of the initial volume. The endpoint of the study was defined as survival at 120 d after treatment.

### Statistical Analysis

On the basis of our previous experience, we selected sample sizes of 3–5 mice for the imaging studies. All other experiments had a minimum sample size of 5, which was enough to detect an effect size of 2 in mean survival with 90% power at a 5% significance level. Statistical comparisons between individual groups were performed via 2-tailed Student *t* testing, with a *P* value of less than 0.05 considered statistically significant. Survival data were presented as Kaplan–Meier curves, and the groups were statistically compared using the log-rank test. All statistics were performed using GraphPad Prism, version 7.

## RESULTS

### Radiochemistry and Stability

**FIGURE 1.**
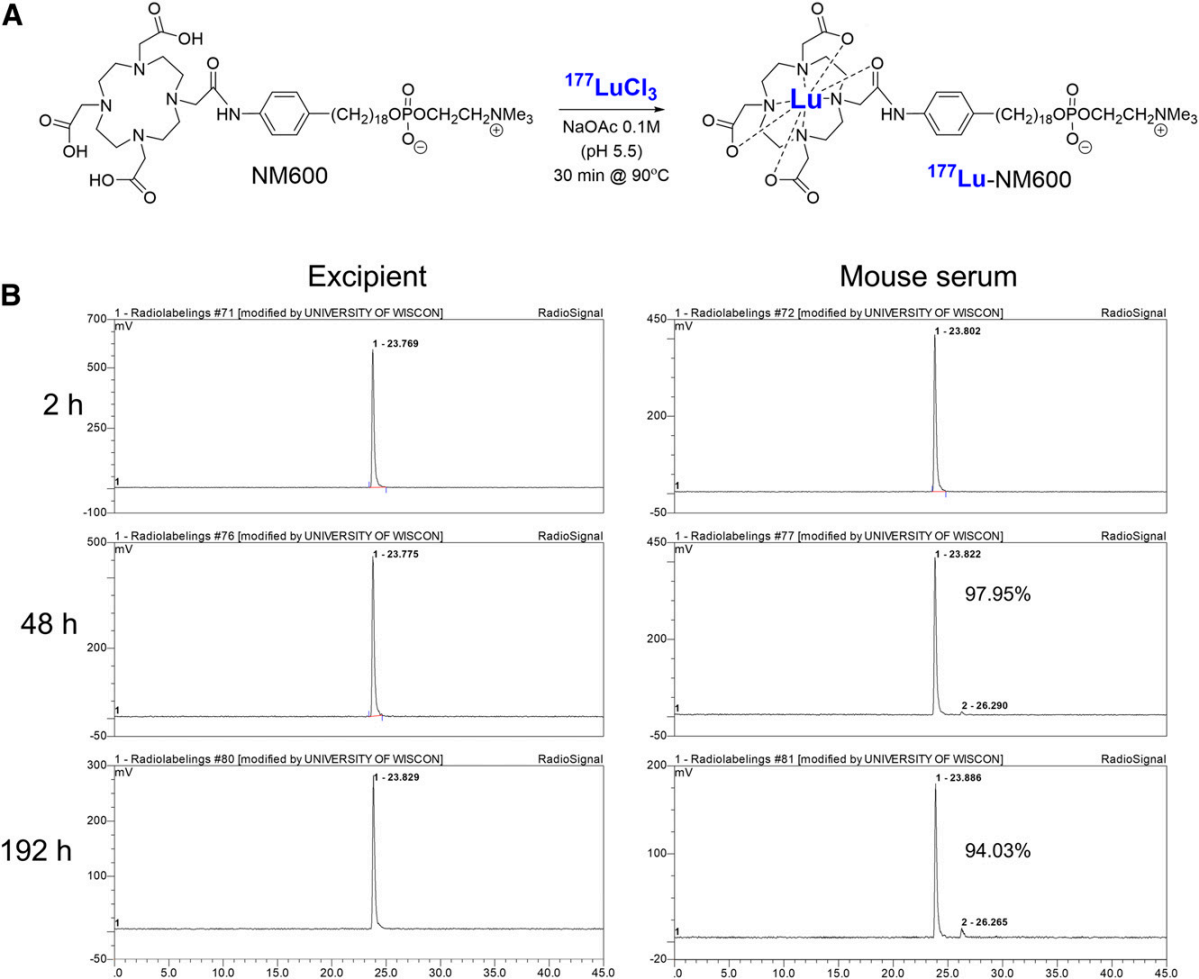
Radiochemical synthesis and stability of ^177^Lu-NM600. (A) Schematic representation of ^177^Lu radiolabeling of NM600. (B) Assessment of ^177^Lu-NM600 stability in injectable formulation and in mouse serum at 37°C for up to 192 h. No degradation was noted when incubated in excipient. Secondary radiopeaks corresponding to impurities were observed in serum, but radiochemical purity remained higher than 94% at 192 h after incubation.

Both ^86^Y-NM600 and ^177^Lu-NM600 were synthesized in nearly quantitative yields (>95%) and with excellent radiochemical purities (>98%), at a similar apparent molar activity of 3.4 MBq/nmol. The stability of ^177^Lu-NM600 in excipient and complete serum was studied by serial radio-HPLC out to 192 h after incubation. No early radiopeaks that might indicate demetallation were noted (Fig. 1A) on HPLC of the excipient; only a single radiopeak (retention time, 23.8 min), corresponding to ^177^Lu-NM600, was observed. At the 192-h time point, a secondary radiopeak (retention time, 26.3 min) was observed in samples incubated in serum, which amounted to 6% of the integrated area of the main radiopeak.

### PET/CT Imaging and Biodistribution

**FIGURE 2.**
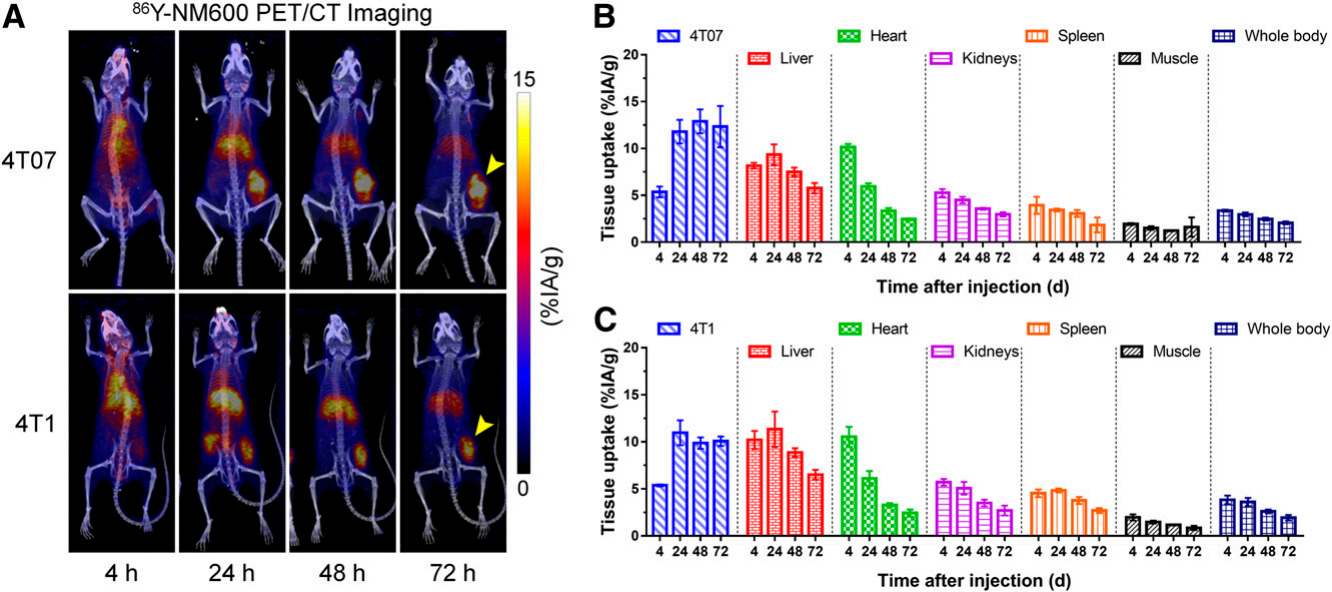
Longitudinal ^86^Y-NM600 PET/CT imaging in murine models of TNBC. (A) PET/CT maximum-intensity projections of mice bearing 4T07 or 4T1 subcutaneous grafts at 4, 24, 48, and 72 h after intravenous administration of ^86^Y-NM600 (*n* = 3). Elevated and persistent uptake of radiotracer in tumor and gradual hepatobiliary clearance were observed. (B and C) Results of quantitative region-of-interest analysis of PET imaging in mice bearing subcutaneous 4T07 (B) or 4T1 (C) breast adenocarcinomas. Data are presented as %IA/g (mean ± SD).

Longitudinal PET/CT studies were performed to investigate the tumor-targeting and biodistribution of ^86^Y-NM600 and to estimate the dosimetry of the ^177^Lu-NM600 therapeutic analog in 2 murine models of breast cancer. Figure 2A shows PET maximum-intensity projections of the in vivo distribution of ^86^Y-NM600 after intravenous injection in BALB/C mice bearing 4T07 or 4T1 tumor grafts. Quantitative region-of-interest analysis of the PET images (Figs. 2B and 2C; Supplemental Table 1; supplemental materials are available at http://jnm.snmjournals.org) revealed elevated blood-pool activity of the radiotracer at the early, 4-h-postinjection, time point, which gradually declined (blood clearance half-life, 20.4 ± 4.3 h) (Supplemental Fig. 1). Because of the hepatobiliary excretion of the agent, peak uptake in the liver at 24 h after injection in the 4T07 and 4T1 models was 9.3 ± 1.1 and 11.3 ± 1.9 %IA/g, respectively, and gradually declined on radiotracer excretion. Uptake in normal organs, including kidneys, spleen, skeletal bones, and muscle, was below 6 %IA/g at all measured time points. Elevated and sustained tumor accretion of ^86^Y-NM600 was observed at 4 h after injection and continued to increase to peak values of 11.0 ± 1.3 and 12.9 ± 1.3 %IA/g in 4T1 and 4T07 tumors, respectively. Prolonged tumor retention was evidenced by a high tumor radioactivity at 72 h after injection.

**TABLE 1:** Summary of Experimental Conditions and Results for ^177^Lu-NM600 Therapy Studies

Tumor model	Treatment arm	Sample size	Injected activity (MBq)	Tumor absorbed dose (Gy)	Median OS (d)
4T07	1	5	Control (0)	0	25
	2	6	9.25	18.9	NA
	3	5	18.5	37.7	NA
4T1	1	10	Control (0)	0	8
	2	7	9.25	15.5	26
	3	10	18.5	31.0	22
	4	7	2 × 9.25	31.0	26
	5	10	18.5 + 9.25	46.5	21

OS = overall survival; NA = not applicable.

**FIGURE 3.**
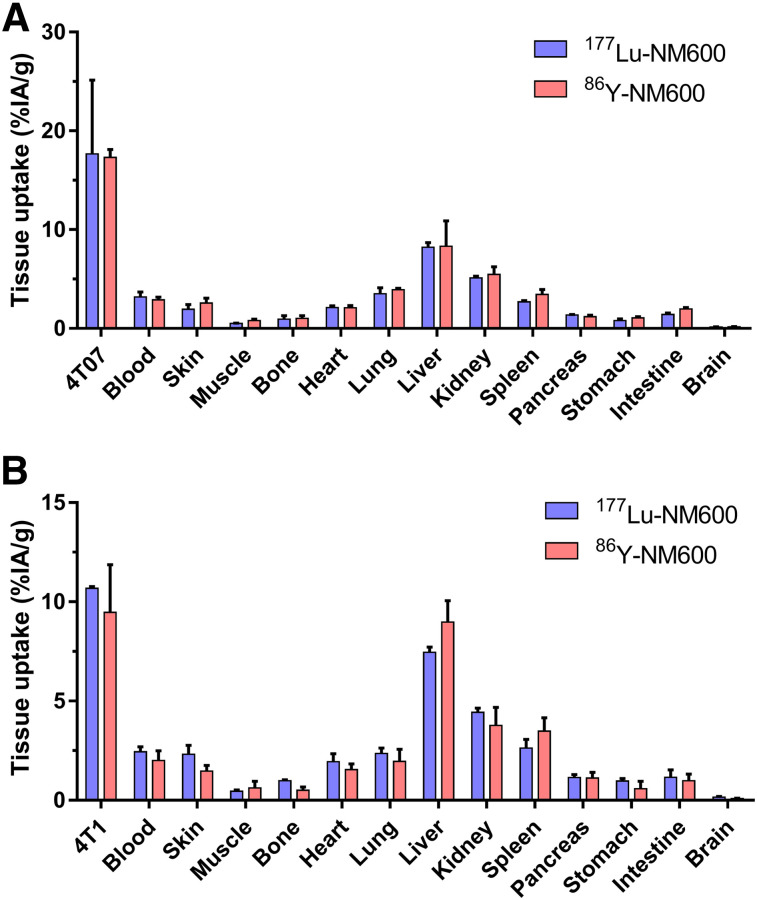
Ex vivo biodistribution of ^86^Y-NM600 and ^177^Lu-NM600 at 72 h after injection in mice bearing 4T07 (A) or 4T1 (B) murine breast cancer grafts. Notable agreement between tumor uptake and normal-tissue distribution of both agents was observed.

After the last imaging session at 72 h after injection of ^86^Y-NM600, ex vivo biodistribution studies were performed and the result compared with similar groups of mice that received ^177^Lu-NM600 (Fig. 3; Supplemental Table 2). Excellent agreement in tumor uptake and normal-tissue biodistribution was observed between ^86^Y-NM600 and ^177^Lu-NM600 injected at the same mass dose, which confirmed the elevated tumor accumulation and retention and the hepatobiliary clearance observed via PET/CT imaging. These results corroborated the feasibility of using ^86^Y as a PET imaging surrogate for ^177^Lu, allowing for the image-based dosimetry estimation of therapeutic ^177^Lu-NM600.

### Dosimetry Estimations

**FIGURE 4.**
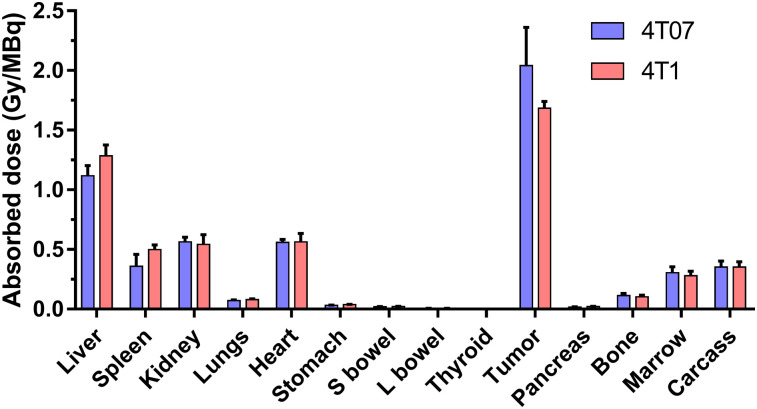
PET/CT imaging–based tumor and normal-organ dosimetry estimation of ^177^Lu-NM600 in BALB/C mice bearing 4T07 and 4T1 breast tumor grafts. Tumors received highest dose of all analyzed tissues, 2.04 ± 0.32 and 1.68 ± 0.06 Gy/MBq for 4T07 and 4T1 tumors, respectively. L = large; S = small.

Tumor and normal-organ dosimetry for ^177^Lu-NM600 in 2 murine tumor models of TNBC were estimated using PET/CT imaging in combination with a standardized mouse model. Figure 4 and Supplemental Table 3 summarize the dosimetry results, showing an absorbed dose per activity of 2.04 ± 0.32 and 1.68 ± 0.06 Gy/MBq to 4T07 and 4T1 tumors, respectively. The normal organ that had the largest dose was the liver, with 1.12 ± 0.09 and 1.28 ± 0.09 Gy/MBq, respectively, which can be attributed to the hepatobiliary clearance of the agent. The remaining normal tissues had absorbed dose values well under 1.0 Gy/MBq, notably the bone marrow, with 0.31 ± 0.05 and 0.28 ± 0.04 Gy/MBq, respectively.

### Toxicity Evaluations

**FIGURE 5.**
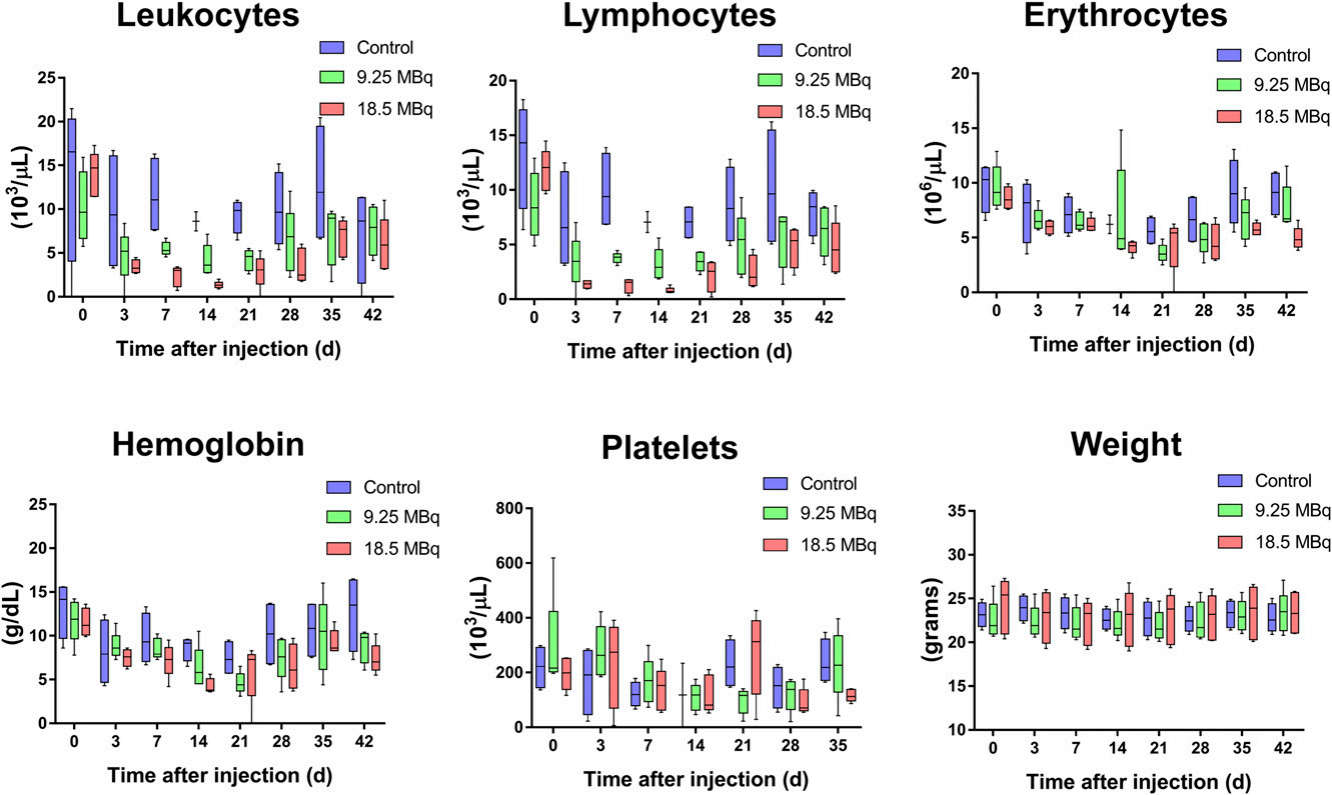
Longitudinal analysis of complete blood counts and weight in mice administered unlabeled NM600 (control) or ^177^Lu-NM600 at 9.25 or 18.5 MBq of injected activity (*n* = 5). Dose-dependent lymphopenia and anemia were observed in treated mice, with nadir at 10 days after injection of agent and 5-wk recovery period. No mortality was reported among treated animals. No significant change in animal weight was noted compared with controls in mice treated with ^177^Lu-NM600.

A longitudinal analysis of complete blood counts in naïve BALB/C mice administered ^177^Lu-NM600 was performed to evaluate potential hematologic toxicity (Fig. 5A). Compared with control mice, injection of 9.25 or 18.5 MBq of ^177^Lu-NM600 resulted in mild, dose-dependent, cytopenia. In both groups, moderate leukopenia was observed, with a nadir at day 10 and resolution at weeks 4 and 5 after treatment in the 9.25- and 18.5-MBq groups, respectively. Anemia was also apparent in treated mice, though to a lesser extent and with faster recovery times. There was no overt thrombocytopenia, as no significant difference in platelet levels between treated mice and controls was observed. Finally, ^177^Lu-NM600 administration had no significant (*P* > 0.05) impact on animal weight.

After a recovery period of 6 wk after ^177^Lu-NM600 administration, histologic (hematoxylin and eosin) examination of the main organs at risk for radiotoxicity, including the liver, kidneys, spleen, bone marrow, and intestine, showed no signs of overt tissue degeneration (Supplemental Fig. 2). The bone marrow presented normal cellularity even at the highest injected activity. Tubular degeneration, an indication of kidney radiotoxicity, was not observed in the kidney sections, and hepatocytes showed normal morphology. Overall, these results corroborated the tolerability of ^177^Lu-NM600 treatment and pointed to the bone marrow as the potential dose-limiting organ.

### ^177^Lu-NM600 Therapy

**FIGURE 6.**
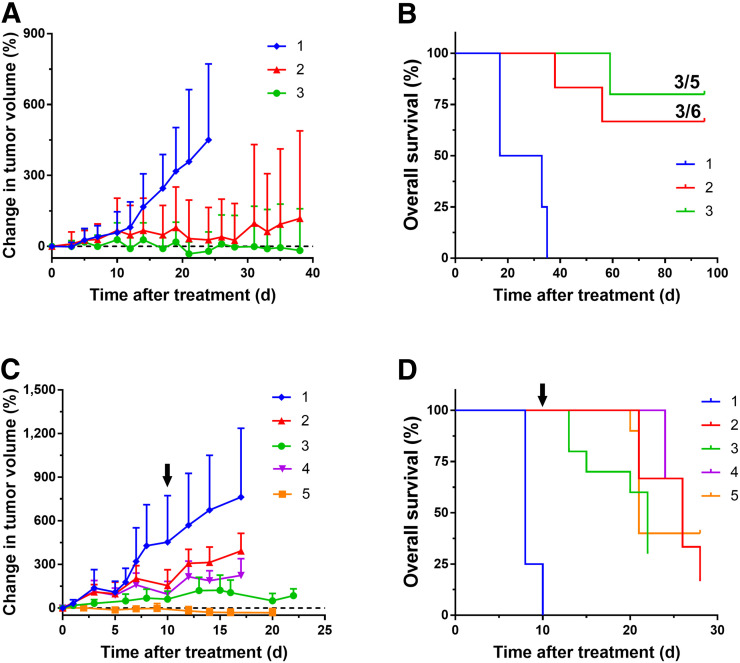
^177^Lu-NM600 treatment inhibits tumor growth and extends survival in mice bearing syngeneic TNBC grafts. (A and B) Tumor growth curves (A) and overall survival (B) of mice bearing 4T07 grafts treated with increasing ^177^Lu-NM600 injected activity. Significant tumor growth inhibition and prolonged median overall survival were noted in all treated mice. Complete response was achieved in 50% (3/6) and 60% (3/5) of mice treated with 9.25 and 18.5 MBq injections, respectively. (C and D) In mice bearing 4T1 tumors, administration of single (9.25 or 18.5 MBq) or fractionated (2 × 9.25 or 18.5 + 9.25 MBq) ^177^Lu-NM600 resulted in significant tumor growth inhibition (C) and extended overall survival (D), although complete responses were not attained. Black arrows denote time of second ^177^Lu-NM600 injection in fractionated treatment arms.

Table 1 summarizes the different treatment cohorts employed in the ^177^Lu-NM600 therapy studies. Three groups of mice (*n* = 5) bearing 4T07 tumor grafts received a single 9.25- or 18.5-MBq injection of ^177^Lu-NM600 or an equimolar amount of nonradioactive NM600. Significant tumor growth inhibition compared with controls (*P* < 0.05) was noted between days 17 and 19 in all treated cohorts (Fig. 6A; Supplemental Fig. 3), and mice receiving 18.5 MBq achieved marked tumor regression. Such an effective tumor response translated into an extended survival in both treatment cohorts (Fig. 6B). In contrast, with a median overall survival of 25 d for the control group, median overall survival was not achieved over the 100-d observation period. Additionally, 60% and 80% of mice achieved a complete tumor response in the 9.25- and 18.5-MBq groups, respectively.

Consistent with the aggressive phenotype of 4T1 tumors, they proved difficult to cure by either single or fractionated ^177^Lu-NM600 treatment. Local tumor control was afforded by treatment with single or fractionated ^177^Lu-NM600 (Fig. 6C; Supplemental Fig. 4), with the group receiving 18.5 MBq followed by 9.25 MBq 10 days after the first dose achieving significant regression of the primary grafts. All 4 treatment schedules resulted in significantly (*P* < 0.0001) prolonged survival compared with controls (Fig. 6D), who quickly succumbed to primary tumor growth. Median overall survival was 8, 26, 22, 26, and 21 d in the control (*n* = 10), 9.25-MBq (*n* = 7), 18.5-MBq (*n* = 10), 2 × 9.25-MBq (*n* = 7), and 18.5 + 9.25-MBq (*n* = 10) groups, respectively. Despite the effective local tumor control and clear survival benefit of ^177^Lu-NM600 treatment, no complete responses were recorded in any of the treatment arms. Necropsy of these mice revealed a prevalent metastatic burden in the lungs, this being the likely cause of death.

## DISCUSSION

Targeted radionuclide therapy has proven effective in patients with advanced, hormone therapy–resistant solid tumors, including metastatic castration-resistant prostate tumors and gastroenteropancreatic neuroendocrine tumors (12,13,18,19), and it is plausible that similar responses can be achieved in TNBC using tumor-selective radiolabeled agents. Therefore, we investigated the properties of ^177^Lu-NM600 as a targeted radionuclide therapy agent for TNBC. Leveraging the radiochemical versatility of NM600, we implemented a theranostic approach using ^86^Y-NM600 as a PET imaging surrogate that estimated the ^177^Lu-NM600 dosimetry and established the dose–effect relationships of the agent.

^86^Y-NM600 was selectively retained in 2 murine models of TNBC. Hepatobiliary excretion of the agent was confirmed by gradual declines in liver and intestinal signals. Overall, ^86^Y-NM600 showed a favorable tissue biodistribution suggesting the existence of a suitable therapeutic window for ^177^Lu-NM600 targeted radionuclide therapy. Both in vivo imaging and ex vivo biodistribution studies validated ^86^Y-NM600 as an imaging surrogate for ^177^Lu-NM600, permitting estimation of ^177^Lu-NM600 dosimetry using PET imaging.

In the 4T07 and 4T1 models, tumor doses were 1.8 and 1.3 times higher, respectively, than doses to the liver, the organ with the highest ^86^Y-NM600 uptake. As predicted by image-derived dosimetry, no overt radiotoxicity was observed by histopathology in the liver, spleen, or kidneys of BALB/C mice administered up to 18.5 MBq of ^177^Lu-NM600, the equivalent to a maximum of 25, 9, and 10 Gy to the liver, spleen, and kidneys, respectively. Bone marrow, on the other hand, showed signs of a dose-dependent radiotoxicity as evidenced by reported transient lymphopenia and anemia after 9.25 or 18.5 MBq of ^177^Lu-NM600 administration. These findings were confirmed by both complete blood counts and histopathology analysis and suggest bone marrow as the dose-limiting organ. Overall, besides mild cytopenia, ^177^Lu-NM600 treatment was well tolerated. Absorbed doses to tumors, which were higher than in any other tissue, were as high as 31 and 38 Gy for a single 18.5-MBq fraction in the 4T07 and 4T1 tumors, respectively. Such elevated tumor doses resulted in effective tumor growth inhibition and prolonged overall survival in all targeted radionuclide therapy arms, compared with NM600-treated ptcontrols. In mice bearing 4T07 tumors, complete tumor eradication was achieved in 50% and 60% of the animals in the 9.25- and 18.5-MBq cohorts, respectively. Despite regression of the primary grafts and extended survival, complete responses were not observed in treated mice bearing the metastatic 4T1 tumors. Use of a fractionated schedule did not additionally benefit survival of the animals, all of whom succumbed to metastatic disease in the lungs.

The failure of ^177^Lu-NM600 to completely stop metastatic progression in 4T1 mice may be ascribed to several conditions. It is likely that at the time of treatment, metastatic tumor foci or large cell clusters (<1–2 mm^3^) had not formed appropriate vasculature, limiting ^177^Lu-NM600 uptake (20,21). Additionally, because of the intermediate range of ^177^Lu β-emissions (1.7 mm), even those metastatic lesions with prominent tumor uptake received a much lower radiation dose than those in a macroscopic tumor with significant cellular crossfire, resulting in tumor progression of the metastatic lesions (22). In follow-up studies, we will investigate the ability of NM600 radiolabeled with short-range, high–linear-energy-transfer radionuclides (e.g., ^225^Ac, ^227^Th, and ^212^Pb) to eradicate microscopic disease. The described microdosimetric effects will be less relevant in human subjects since metastatic breast cancer lesions tend to be much larger at diagnosis in humans (>5 mm) than in mice (<1 mm) (23). Finally, another approach to increase the cure rate in this aggressive model will be to combine ^177^Lu-NM600 with other systemic antitumor therapies, such as immunotherapy or poly(adenosine diphosphate-ribose) polymerase inhibitors, which have shown promise in patients carrying BRCA1 and BRCA2 mutations (12,13,18,19). The potential of this approach was demonstrated by Filatenkov et al. (24) using external-beam radiotherapy in combination with adoptive T-cell transplantation to achieve long-term survival in the 4T1 model.

The absorbed doses based on our image-based dosimetry are likely to be underestimated because of the limitations associated with small-animal PET imaging. Because correction for positron range was not performed, partial-volume effects may have contributed to uncertainty in the dosimetry results. For example, the relatively long positron range of ^86^Y (mean, 3.9 mm) can cause signal spillover into organs adjacent to the liver, potentially leading to an overestimation of activity and dose within these organs. Additionally, because of the relatively small β-energy of ^177^Lu (average pathlength, <1 mm), there is minimal dose spillover between organs that are not in direct contact with one another. Therefore, any cross-fire between organs is mainly due to partial-volume effects innate to the PET imaging and not the energy of the β-particles. These problems associated with the image acquisition will be less relevant as we progress to human studies, in which the spacing between neighboring organs is larger than the positron range of ^86^Y.

## CONCLUSION

We demonstrated that ^177^Lu-NM600 may be a promising treatment option for TNBC by extending survival in a population of patients for whom effective therapies are lacking. The favorable safety profile of ^177^Lu-NM600, and its efficacy against aggressive tumor models, provide motivation for continual investigation and potential clinical translation.

## DISCLOSURE

Reinier Hernandez, Joseph Grudzinski, and Anatoly Pinchuk are consultants to Archeus Technologies, Inc. Jamey Weichert is a cofounder and stockholder of Archeus Technologies, Inc., which holds the licensing rights to NM600. The University of Wisconsin Carbone Cancer Center provided support grant P30 CA014520 and gifts. Part of this work was supported by the National Cancer Institute of the National Institutes of Health (awards U01CA233102 and T32CA009206). No other potential conflict of interest relevant to this article was reported.

**KEY POINTS**

**QUESTION:** Is targeted radionuclide therapy a viable treatment option for TNBC?
**PERTINENT FINDINGS**: In 2 murine models of locally advanced and metastatic TNBC, we demonstrated the ability of ^177^Lu-NM600 to selectively deliver significant radiation doses to tumors. Such high cumulative absorbed doses resulted in significant tumor control and extended survival without incurring prohibitive normal-tissue toxicity.
**IMPLICATIONS FOR PATIENT CARE:** In this aggressive cancer type with few treatment options, ^177^Lu-NM600 has the potential to become a clinically viable therapeutic agent for TNBC.
