# Supplementary material for: 177Lu-NM600 Targeted Radionuclide Therapy Extends Survival in Syngeneic Murine Models of Triple-Negative Breast Cancer
Source: J Nucl Med. 2020 Aug;61(8):1187–94. doi: 10.2967/jnumed.119.236265 (PMC7413241; doi:10.2967/jnumed.119.236265)
Supplement: Supplementary file 1 [file jnm236265SupplementaryData.pdf]

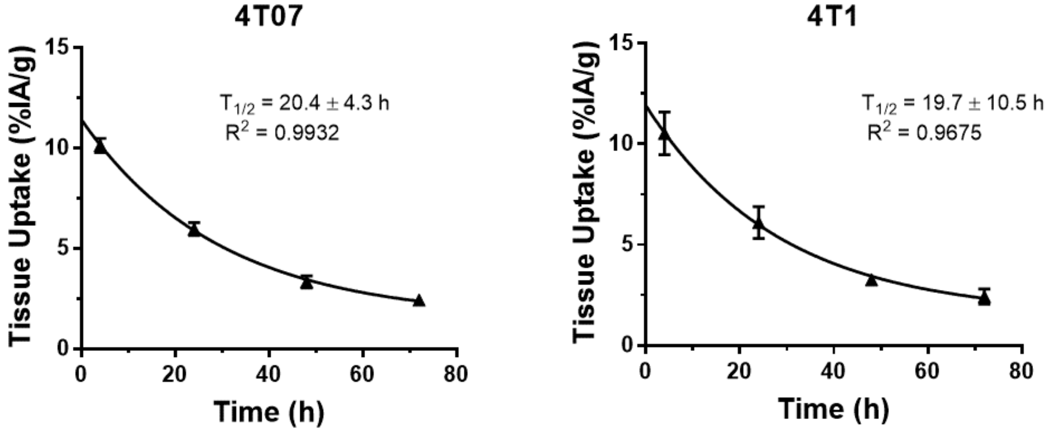

Supplemental figure 1 Blood time-activity curves of ^{86}Y-NM600 in Balb/C mice bearing syngeneic s.c. 4T07 or 4T1 breast tumors. Blood circulation half-life ($T_{1/2}$) was calculated by fitting the quantitative PET data to a monoexponential decay. Similar circulation $T_{1/2}$ were observed (20.4 ± 4.3 h vs. 19.7 ± 10.5 h) between the two groups.

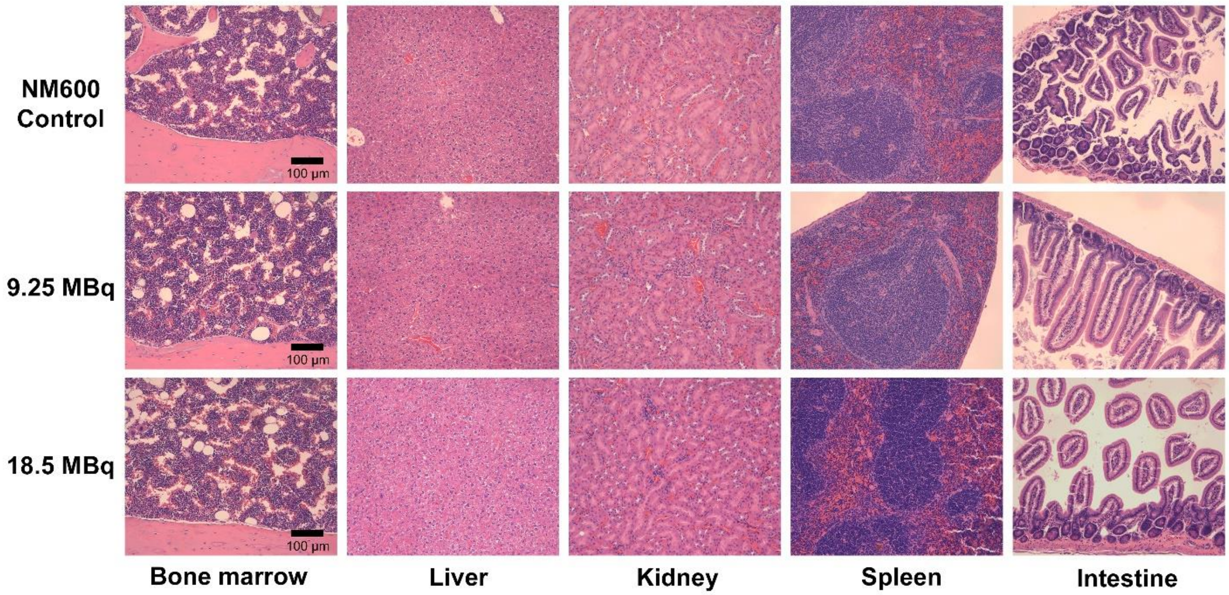

Supplemental figure 2. Histopathology examination of H&E tissue sections corresponding to the bone marrow, liver, kidney, spleen, and small intestine, six weeks after injection of NM600, or ^{177}Lu-NM600 9.25 MBq or 18.5 MBq. Similar tissue morphology was noted in all analyzed sections, indicating the absence of tissue degeneration. Scale bars represent 100 μm

Supplemental table 1 Results of the region-of-interest analysis of the longitudinal PET/CT data in mice bearing 4T1 or 4T07 subcutaneous grafts and administered ^{86}Y-NM600 (9.25 MBq). Data are presented as %IA/g (mean ± SD).

Time (h p.i.)	Tumor (%IA/g)		Heart/blood (%IA/g)		Liver (%IA/g)		Spleen (%IA/g)		Kidneys (%IA/g)		Muscle (%IA/g)		Bone/Marrow (%IA/g)		Whole Body (%IA/g)	
	**Mean**	**SD**	**Mean**	**SD**	**Mean**	**SD**	**Mean**	**SD**	**Mean**	**SD**	**Mean**	**SD**	**Mean**	**SD**	**Mean**	**SD**
**86Y-NM600 in 4T1 bearing mice**																
**4**	5.4	0.1	10.5	1.1	10.2	1.0	4.5	0.4	5.7	0.4	2.0	0.3	2.2	0.3	3.8	0.5
**24**	11.0	1.3	6.1	0.8	11.3	1.9	4.8	0.2	5.1	0.7	1.5	0.2	1.7	0.3	3.6	0.4
**48**	9.9	0.6	3.3	0.2	8.9	0.5	3.8	0.4	3.5	0.4	1.2	0.1	1.3	0.1	2.6	0.2
**72**	10.1	0.5	2.4	0.4	6.5	0.5	2.7	0.3	2.7	0.5	0.8	0.2	1.0	0.1	1.9	0.3
**86Y-NM600 in 4T07 bearing mice**																
**4**	5.4	0.6	10.1	0.4	8.2	0.3	3.9	0.9	5.3	0.4	1.9	0.1	2.2	0.1	3.4	0.1
**24**	11.8	1.3	6.0	0.3	9.3	1.1	3.4	0.2	4.5	0.3	1.5	0.2	1.8	0.3	3.0	0.2
**48**	12.9	1.3	3.3	0.3	7.5	0.5	3.1	0.4	3.6	0.1	1.2	0.1	1.4	0.3	2.5	0.2
**72**	12.3	2.2	2.4	0.1	5.8	0.6	1.8	0.8	3.0	0.2	1.6	1.0	1.1	0.2	2.1	0.2

Supplemental table 2 Results of *ex vivo* biodistribution in mice bearing 4T07 or 4T1 tumors grafts, and administered either 86Y-NM600 (9.25 MBq) or ^{177}Lu-NM600 (9.25 MBq), 72 h post-injection. Data is presented as %IA/g (mean ± SD).

	4T07 bearing mice				4T1 bearing mice			
Agent	86Y-NM600		^{177}Lu-NM600		86Y-NM600		^{177}Lu-NM600	
**Tissue**	**Mean***	**SD**	**Mean***	**SD**	**Mean***	**SD**	**Mean***	**SD**
Tumor	17.31	0.80	17.67	7.48	9.47	2.40	10.67	0.08
Blood	2.89	0.27	3.19	0.51	2.01	0.49	2.45	0.25
Skin	2.56	0.51	1.94	0.48	1.47	0.28	2.32	0.44
Muscle	0.78	0.17	0.52	0.02	0.62	0.35	0.46	0.05
Bone/Marrow	1.02	0.27	0.94	0.34	0.51	0.16	0.98	0.05
Heart	2.09	0.23	2.12	0.18	1.55	0.29	1.94	0.41
Lung	3.91	0.16	3.50	0.61	1.95	0.61	2.36	0.27
Liver	8.31	2.56	8.21	0.47	8.98	1.08	7.45	0.27
Kidney	5.45	0.79	5.10	0.19	3.77	0.91	4.44	0.21
Spleen	3.44	0.50	2.70	0.11	3.48	0.68	2.64	0.43
Pancreas	1.19	0.14	1.37	0.06	1.12	0.28	1.15	0.15
Stomach	1.07	0.12	0.8	0.17	0.58	0.38	0.97	0.12
Intestine	1.96	0.17	1.41	0.15	0.99	0.33	1.15	0.39
Brain	0.17	0.02	0.13	0.04	0.08	0.03	0.16	0.03

*****data is presented as percent injected activity per gram of tissue (%IA/g)

Supplemental table 3 Summary of results from the dosimetry estimation for ^{90}Y-NM600 using longitudinal ^{86}Y-NM600 PET/CT data and The Standard Mouse Model.

Target Organ	Cumulative Activity [(MBq-sec)/MBq]	$\sum_s \boldsymbol{\phi_B(r_t \leftarrow r_s)(\tilde{A}_s)}$	Absorbed Dose (Gy/MBq)	Dose (9.25 MBq)
**Liver**	28644.57	21282.91	3.04	28.1
**Spleen**	1332.53	1044.35	1.74	16.1
**Kidney**	3980.59	3058.51	1.73	16.0
**Lungs**	1361.76	3344.07	3.34	30.9
**Heart**	1943.09	1451.62	1.89	17.5
**Stomach**	1588.72	2322.27	1.99	18.4
**S bowel**	7970.82	10947.58	1.87	17.3
**L bowel**	3014.02	2799.23	1.26	11.7
**Thyroid**	953.23	963.16	1.37	12.7
**Tumor**	26004.45	17978.74	2.69	24.9
**Pancreas**	953.23	662.53	0.95	8.7
**Bone**	998.62	1041.73	1.42	13.1
**Marrow**	30.80	47.16	0.79	7.3
**Carcass**	191699.11	194828.76	1.38	12.8

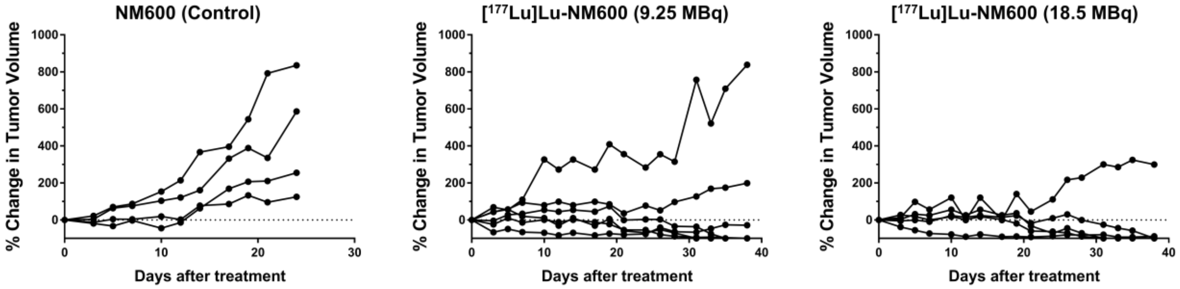

Supplemental figure 3 Individual weight of mice bearing s.c. 4T07 tumors injected with unlabeled NM600, or 9.25 or 18.5 MBq of ^{90}Y-NM600. Data are presents as percent of the initial weight.

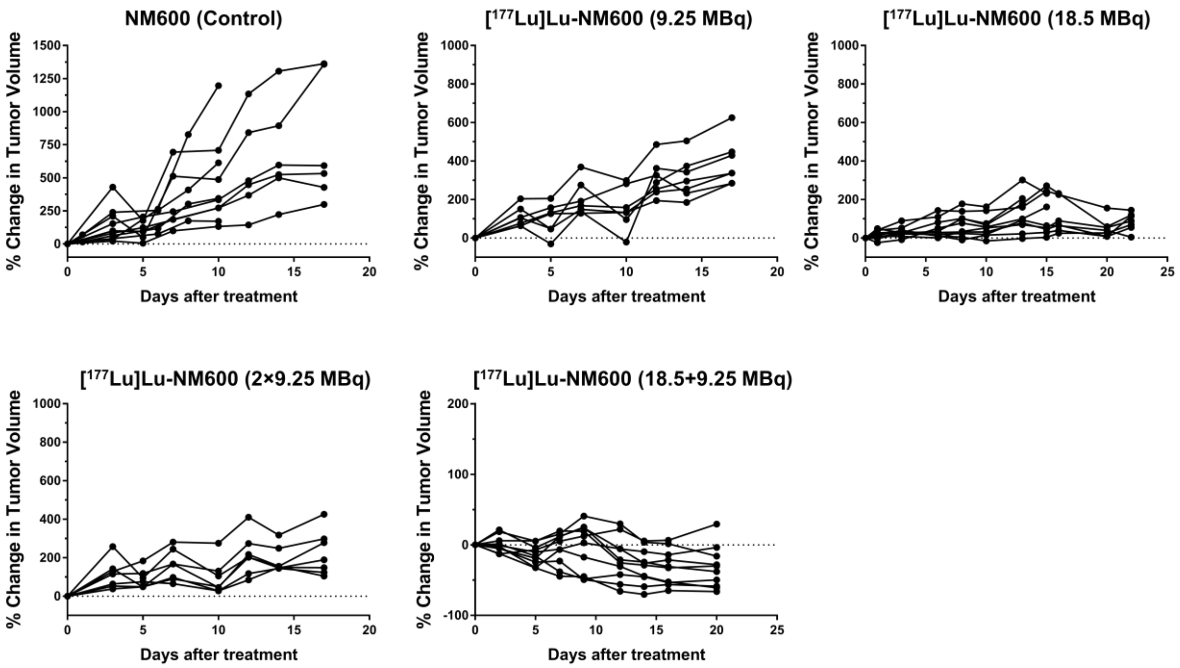

Supplemental figure 4 Individual tumor growth curves of mice bearing s.c. 4T1 tumors injected with unlabeled NM600, 9.25, 18.5, 2×9.25, or 18.5 + 9.25 MBq of ^{177}Lu-NM600. Data are presented as a fraction of the initial tumor volume.
